# Supplementary material for: Forced intercourse in America: a pandemic update
Source: BMC Public Health. 2023 Jun 21;23:1201. doi: 10.1186/s12889-023-16102-y (PMC10283174; doi:10.1186/s12889-023-16102-y)
Supplement: Supplementary file 1 — Supplementary Material 1 [file 12889_2023_16102_MOESM1_ESM.pdf]

Additional File

eTable 1. Pre-Pandemic to Early Pandemic Changes in Sexual Activity in the U.S. General Population

	Proportion of Women Sexually Active in the Past 12 Months[a]		
	**NSFG 2015-2017**	**NSFG 2017-2019**	**AFHS 2020-2021**
Ages 18-22	.80 (.02)	.71 (.03)	.48 (.06)**
Ages 23-28	.85 (.02)	.84 (.01)	.78 (.05)
Ages 29+	.89 (.01)	.87 (.01)	.81 (.03)*

[a] Values in parentheses are design-adjusted standard errors (SE), and all estimates are weighted. AFHS is different from other samples at *$p < 0.05$ or ** $p < 0.01$ (based on design-adjusted chi-square tests).
